# T Cells in Atherosclerosis: Key Players in the Pathogenesis of Vascular Disease

**DOI:** 10.3390/cells12172152

**Published:** 2023-08-26

**Authors:** Hannah Hinkley, Daniel A. Counts, Elizabeth VonCanon, Michael Lacy

**Affiliations:** Department of Medical Laboratory Sciences, Virginia Commonwealth University, Richmond, VA 23298, USA

**Keywords:** T cells, atherosclerosis, inflammation, cardiovascular disease

## Abstract

Atherosclerosis is a chronic inflammatory disease characterized by the accumulation of lipid-rich plaques within arterial walls. T cells play a pivotal role in the pathogenesis of atherosclerosis in which they help orchestrate immune responses and contribute to plaque development and instability. Here, we discuss the recognition of atherosclerosis-related antigens that may trigger T cell activation together with additional signaling from co-stimulatory molecules and lesional cytokines. Although few studies have indicated candidates for the antigen specificity of T cells in atherosclerosis, further research is needed. Furthermore, we describe the pro-atherogenic and atheroprotective roles of diverse subsets of T cells such as CD4^+^ helper, CD8^+^ cytotoxic, invariant natural killer, and γδ T cells. To classify and quantify T cell subsets in atherosclerosis, we summarize current methods to analyze cellular heterogeneity including single cell RNA sequencing and T cell receptor (TCR) sequencing. Further insights into T cell biology will help shed light on the immunopathology of atherosclerosis, inform potential therapeutic interventions, and pave the way for precision medicine approaches in combating cardiovascular disease.

## 1. Introduction

Atherosclerosis, a chronic inflammatory disease of the arterial wall, is the primary pathology underlying cardiovascular disease (CVD), which remains a leading cause of morbidity and mortality worldwide [1,2]. The development and progression of atherosclerosis involves a complex interplay of various immune cells and molecular pathways [3]. While the role of macrophages and lipid deposition has traditionally garnered significant attention [4,5], emerging evidence highlights the contribution of T cells in orchestrating the inflammatory milieu within atherosclerotic plaques [6,7]. In particular, recent evidence in pre-clinical and clinical models of atherosclerosis indicate T cells may represent the majority of lesional immune cells [7,8].

The infiltration of immune cells, including T cells, plays a key role in the initiation and progression of atherosclerosis. Following damage to endothelial cells and lipid deposition, T cells can loosely attach to the endothelium using selectins such as L-selectin and firmly attach through various integrins [9,10]. Chemokine gradients, which have been shown to be upregulated in atherosclerotic plaques [11], can signal through C-C motif chemokine receptors (CCRs) and C-X-C motif chemokine receptors (CXCRs) as well as their respective ligands to recruit T cells as well as other immune cells, including neutrophils, macrophages, B cells, dendritic cells, as well as others, into lesions [12]. As described in subsequent sections, once T cells infiltrate plaques, they may clonally expand and secrete inflammatory cytokines, ultimately leading to plaque instability and rupture [13,14].

Here, we focus on the current understanding of T cell biology in atherosclerosis, with a particular focus on their activation pathways as well as individual T cell subset contributions within the context of plaque progression. We will explore recent data on the impact of metabolic and environmental factors on T cell responses. Additionally, we aim to describe current methods, including novel transcriptomic and proteomic methods, to quantify and elucidate the role of T cells in mouse and human atherosclerotic plaques.

## 2. Factors Influencing T Cell Activation

To initiate activation, T cells need to be presented antigenic peptides. Antigens are first digested into smaller peptide fragments, then loaded onto specialized surface proteins called major histocompatibility complexes (MHC) to be delivered to naïve T cells. For antigen presentation, there are two classes of MHC proteins, class one (MHC-I) and class two (MHC-II), characterized by their specific cellular functions. In particular, MHC-I proteins are expressed by all nucleated cells and present antigenic peptides derived from the intracellular breakdown of endogenous antigens to CD8^+^ cytotoxic T cells [15]. On the other hand, MHC-II proteins are found primarily on the surface of antigen-presenting cells (APCs) and present peptides derived from the phagocytosis of extracellular antigens to CD4^+^ T cells [16]. In a mouse model of atherosclerosis, loss of MHC-II antigen presentation in hyperlipidemic *ApoE* deficient mice with a genetic *MHCII* deficiency led to a lack of splenic CD4^+^ T cells while double negative (DN) and CD8^+^ T cells increased, which ultimately led to a 2-fold increase in atherosclerosis in the aortic root. The authors contributed this increase in atherosclerosis to a loss of CD4^+^ T regulatory (Treg) cells, which have been associated with an atheroprotective role [17].

### 2.1. Cognate Antigen Recognition

Several studies have investigated associations between *MHC* genes, also known as human leukocyte antigen (HLA) genes, and the severity of atherosclerosis. Within MHC-I, alleles for HLA-B*13:02 and HLA-C*06:02 were associated with a higher atherosclerotic burden while HLA-B*38:01 was associated with less atherosclerosis when researchers adjusted for other cardiovascular risk factors in patients with psoriatic disease [18]. Within MHC-II, the HLA-DRB1 allele has been associated with cardiovascular events in rheumatoid arthritis and polyarthritis patients [19,20]. These results imply that high inflammatory states like those observed in autoimmune arthritic diseases may be explained by particular alleles for antigen presentation, and this high inflammation from the adaptive immune system can contribute to cardiovascular events and mortality as well (see Table 1).

After antigenic peptides are loaded and presented on surface MHC molecules, the MHC-peptide complex joins a surface T cell receptor (TCR) of a naïve T cell. As expected, mouse and human models of atherosclerosis have demonstrated antigen-experienced T cells, which upregulate the surface marker CD44 [21,22]. More recently, TCR-sequencing studies revealed the possibility of specific atherosclerosis-antigens due to clonal T cell expansion [23,24], which we discuss further in subsequent sections. Although the effector functions of T cells are dependent in part on the antigen presented, the specificity of atherosclerotic antigens is not entirely known. The most probable atherosclerosis-antigen is the low density lipoprotein (LDL) and its associated molecules, including its oxidized form, oxLDL, as well as its core protein, Apolipoprotein B [25,26,27]. In addition to LDL, heat shock proteins (HSP), such as HSP60 and HSP70, have been linked to the progression of atherosclerosis [28,29].

### 2.2. Co-Stimulation

After cognate antigen recognition, T cells require co-stimulation to become fully activated. An immunological synapse forms on the surface of T cells following antigen presentation where co-stimulatory and co-inhibitory surface proteins are rearranged to be near the TCR [30]. Considering its role in T cell activation, several studies have examined the downstream effect of co-stimulation on the adaptive immune system’s role in atherosclerosis. The expression of CD80 and CD86, co-stimulatory markers typically observed on dendritic cells which stimulate T cells through CD28, has been associated with atherosclerotic plaque inflammation. A combined genetic *Cd80*/*Cd86* deficiency revealed a lowered atherosclerotic burden in early atherosclerosis, which may be attributed to a reduction in lesional APCs as well as a T helper (T_h_) 1 response [31]. However, a separate study found that *Cd80*/*Cd86* deficiency reduced numbers of natural T regulatory (Treg) cells and led to increased atherosclerosis following 20 weeks of a high-fat, high-cholesterol diet [32]. Similarly, the expression of CD40, found on APCs, and CD40L, expressed on T cells, has been linked to atherosclerosis as well as plaque instability [33,34,35]. The disruption of the CD40-CD40L dyad in hyperlipidemic mice suppressed T_h_1 responses, particularly interferon (IFN)-γ expression, and reduced plaque burden [36]. Additional co-stimulatory molecules such as the glucocorticoid-induced tumor necrosis factor receptor (GITR) [37], CD27 [38], and programmed cell death protein 1 (PD1) [39] have demonstrated effects on adaptive immunity and T cell phenotypes in atherosclerosis (see Table 1).

### 2.3. Metabolism

Metabolism can play a crucial role in the activation as well as the polarization of T cells. Before activation, naïve T cells primarily utilize oxidative phosphorylation (OXPHOS) for energy production, but a western diet has been shown to reduce their proliferative capacity in hyperlipidemic mice compared to a standard diet due to disruption of glycolysis [40,41]. Similarly, in humans, naïve T cell numbers are reduced in patients with more severe CVD, which may be attributed to a reduced proliferative capacity as well [22,40]. After activation, effector T cells switch to glycolysis as their primary metabolic pathway. Glucose transporter-1 (GLUT1) receptors, which import glucose to convert into pyruvate, are upregulated on the surface of T helper cell subsets [42]. However, certain subsets may have specific additional metabolic requirements, such as de novo fatty acid synthesis in T_h_17 as observed under conditions that inhibit Acetyl-CoA carboxylase [43].

Tregs, on the other hand, rely less on glycolysis and more on lipid metabolism and OXPHOS to sustain their atheroprotective effects in cardiovascular disease [44]. Migration of activated Tregs to inflamed tissue has been shown to be dependent on glycolysis, which may have similar implications on the endothelium during atherosclerotic plaque progression [45]. However, increased glycolysis also led to a decreased expression of the signature Treg transcription factor, forkhead box P3 (FoxP3), suggesting those Tregs may lose their suppressive capacity [46]. On the other hand, AMP-activated protein kinase (AMPK), which can be directly activated by liver kinase B1 (LKB1), limits glycolysis and encourages OXPHOS by inhibiting enzymes necessary for the TCA cycle. Mice with LKBI-deficient Tregs resulted in the development of severe autoimmune disease. Furthermore, many of the LKBI-deficient Tregs began to produce pro-inflammatory cytokines, such as IFN-γ usually associated with effector T cells [47]. In the context of atherosclerosis, the in vitro incubation of oxidized lipoproteins such as oxLDL with Tregs reduced their suppressive capacity, likely through the downregulation of FoxP3 [48]. Using a hyperlipidemic *Ldlr*^−/−^ mouse model with fluorescently-labeled Tregs, it was demonstrated that Tregs initially increase in atherosclerotic lesions but ultimately decline during prolonged hypercholesteremia, which may be explained by increased Treg plasticity resulting in the downregulation of traditional Treg expression patterns and increased T_h_1-like expression [49,50].

### 2.4. Additional Factors Influencing T Cell Activation

T cell activation, proliferation, and metabolism have been shown to have profound effects in murine models of atherosclerosis. To mimic cholesterol accumulation during T cell aging, hyperlipidemic mice with a T cell-specific ATP-binding cassette A1 and G1 (ABCA1/ABCG1) deficiency increased T cell apoptosis and senescence leading to reduced plaque burden [51]. Dietary supplementation with the amino acid homoarginine also reduced atherosclerosis in hyperlipidemic mice, which was attributed to reductions in T cell proliferation, migration, and expression of inflammatory cytokines [52]. Metabolic reprogramming via epigenetic enzymes can also affect T cell differentiation and the lesional cytokine environment as in the case of the histone 3 lysine 27 (H3K27) demethylase, Jumonji domain-containing protein-3 (JMJD3), which regulates T_h_17 differentiation [53]. We discuss the effects of individual T cell subsets on atherosclerosis in the following section.

## 3. Pro-Atherogenic and Atheroprotective T Cell Subsets

**Figure 1:**
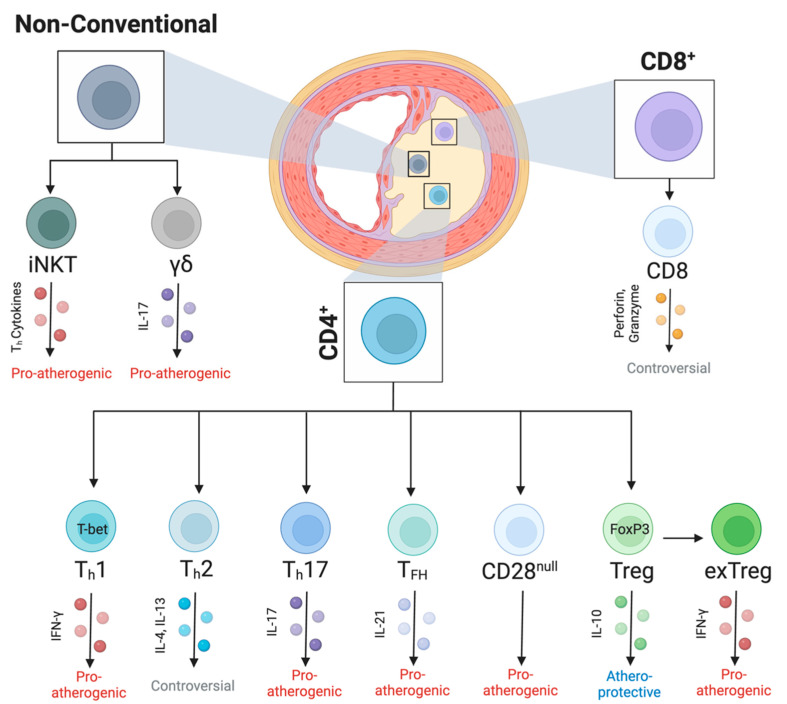
Role of T cell subsets in atherosclerosis. Lesional T cells can be broken up into three groups including T cells with α and β chains within their T cell receptor (TCR) like CD4^+^ T cells (middle) and CD8^+^ T cells (right) as well as non-conventional T cells that follow different activation pathways (left). Non-conventional T cells include the pro-atherogenic invariant natural killer T (iNKT) cells that express various T helper (T_h_) cytokines as well as interleukin (IL)-17 expressing γδ T cells. CD4^+^ T cells include T_h_ subsets such as pro-atherogenic T_h_1 that express interferon-γ, controversial T_h_2 that expresses IL-4 and IL-13, and T_h_17 that expresses IL-17. Follicular T helper (T_FH_) cells as well as CD28^null^ T cells are both CD4^+^, which are pro-atherogenic. Additionally, regulatory T (Treg) are atheroprotective CD4^+^ T cells; however, in late stage atherosclerosis, they convert to pro-atherogenic ex-Tregs. Finally, CD8^+^ T cells can infiltrate atherosclerotic plaques as well, but their role remains unclear. Created with biorender.com.

Following activation, T cells can differentiate into various pro-atherogenic or atheroprotective subsets classified based on their specific expression of transcription factors and cytokines. Within atherosclerotic lesions, a range of T cell subsets can be found; however, CD4^+^ T cells and their various helper T cell subsets are the most abundant [12] (see Figure 1). As a whole, the depletion of CD4^+^ T cells protects against atherosclerosis [54].

### 3.1. T_H_1

Within the lesional T helper cell subsets, T_H_1 cells are the predominant T cell subtype found in both human and mouse atherosclerotic plaques [12]. It is widely believed that T_H_1 cells promote inflammation, plaque instability, and lesion growth within atherosclerosis [55]. Genetic deficiency of the T_H_1 lineage-specific transcription factor, TBX21 (T-bet), as well as genetic deficiency of IFN-y reduced atherosclerosis in hyperlipidemic mice, suggesting a pro-atherogenic role [56,57]. Furthermore, studies have indicated that IFN-y may inhibit vascular smooth muscle cell (VSMC) proliferation, which can weaken the fibrous cap of the plaque and lead to rupture [58].

### 3.2. T_H_2

Following T_H_1 cells, T_H_2 cells are the next most abundant T cell subgroup in atherosclerotic lesions [12]. T_H_2 cells are typically thought to be involved in immune responses in cases of parasites, tissue damage, asthma, and other allergic reactions; however, many T_H_2 cell cytokines, including IL-4 and IL-13, are found in mouse atherosclerotic lesions. Yet, whether T_H_2 cells are considered atheroprotective or pro-atherogenic remains largely controversial.

IL-4 is the main T_H_2 cytokine and has been studied in-depth to determine its role in atherosclerotic plaques. In some studies, T_H_2 cytokines have been shown to exhibit pro-atherogenic properties in mice. Researchers crossbred IL-4 deficient mice (*IL-4*^−/−^) with hyperlipidemic, apolipoprotein E-deficient (*ApoE*^−/−^) mice to investigate the role of T_H_2 cells in atherogenesis [59]. At 45 weeks, *ApoE*^−/−^*/IL-4*^−/−^ mice had a significant decrease of 58% and 64% of atherosclerosis located in the aortic arch compared to *ApoE*^−/−^ and *ApoE*^−/−^*/IL-12*^−/−^ mice, respectively. Additionally, there was a 78% reduction in thoracic aortic lesions compared to *ApoE*^−/−^*/IL-12*^−/−^ [59]. These findings suggest that T_H_2 cytokines, specifically IL-4, may contribute to the development of atherosclerosis disease in *ApoE*^−/−^ mice. When exogenous IL-4 was administered in a separate study using hyperlipidemic *Ldlr*^−/−^ mice, however, no changes were observed in atherosclerotic lesions between experimental and control mice [60].

In other studies, IL-4 has been proven to have anti-atherogenic properties. *IL-4*^−/−^ mice were injected with liver-specific AAV expressing Pcsk9 and fed a western diet for 16 weeks to promote the formation of atherosclerotic plaques [61]. This allowed researchers to review the abundance of IL-4-producing cells in plaques. Following the 16-week trial, mice in the resolution of the atherosclerosis group (res) were injected with anti-sense oligonucleotide (ASO) to apolipoprotein B (ApoB) and were fed a normal chow diet. This induced a decrease in total cholesterol to normal levels (60 mg/dL) in the res group. After performing an analysis of res group aortic arches using flow cytometry, no statistical significance was noted in the proportion of IL-4 cytokines. However, the limited number of IL-4 cytokines that accumulate in progressing plaques remained constant during the resolution of the disease, indicating that IL-4 collaborates with other factors in the atherosclerosis resolution, namely, STAT6 and Wnt signaling in macrophages [61].

Unlike the controversial role of IL-4, most studies agree that IL-13 contains anti-atherogenic properties. To study the effect of IL-13 on atherosclerotic lesions, researchers transplanted *LDLr*^−/−^ mice with bone marrow from either *IL-13*^−/−^ or *IL-13*^+/+^ mice and were fed a high-fat diet [62]. In mice that received *IL-13*^−/−^ bone marrow, atherosclerotic lesions showed an increased necrotic core formation and accelerated atherosclerosis compared to *IL-13^+/+^* mice. These findings were consistent with advanced plaque morphology and showed that IL-13 protects from atherosclerosis.

### 3.3. T_H_17

T_H_17 cells are activated by their main cytokine, IL-17. IL-17 is known to play a role in the maintenance of tissues in response to injury, physiological stress, and infection [63]. In the context of atherosclerosis, IL-17 is also believed to play a large role in plaque development. Researchers bred ApoE^−/−^ mice with IL-17A-deficient (*IL17a*^−/−^) and IL-17 receptor A-deficient mice (*IL17ra*^−/−^) to determine the role of the IL-17A cytokine on atherosclerosis, and they observed a significant decrease in aortic plaque formation in *IL17a*^−/−^*/ApoE*^−/−^ mice in comparison to chow-fed *IL17a*^−/−^*/ApoE*^−/−^, *IL17ra*^−/−^*/ApoE*^−/−^ and *ApoE*^−/−^ mice [64]. Researchers also noted that plaque development from the expression of IL-17A is largely dependent on the stage of the lesion as well as the anatomical site of the lesion. Lesions in the aortic arch showed that the release of IL-17A induced the release of chemokines, resulting in monocyte and neutrophil recruitment.

### 3.4. T_FH_

T follicular helper (T_FH_) cells are a subset of CD4^+^ and are required for B-cell activation and differentiation into memory or plasma cells. This can lead to the overactivation and differentiation of B cells and is critically regulated by Qa-1—restricted CD8^+^ Tregs [65]. By blocking the inducible T cell co-stimulator (ICOS) and its ligand (ICOSL) in *Apoe*^−/−^ mice, researchers noted a reduction of T_FH_ cells in tertiary lymphoid organs and a subsequent reduction in atherosclerotic plaques. The major cytokine of T_FH_ cells, IL-21, is also essential for B-cell activation and has been studied to determine its role in atherogenesis. The amount of IL-21 in the adventitia of abdominal aortic aneurysm (AAA) tissue was quantitated and proportional to the AAA’s diameter. This data indicated a strong link to atherogenesis and progression with the T_FH_-dependent B-cell response.

### 3.5. CD28^null^ T Cells

Unlike the previously mentioned CD4^+^ T cells, CD28^null^ T cells are only found in humans and other primates [12]. Therefore, many research studies focus on human correlations, including patients with end-stage renal disease (ESRD) and coronary diseases, such as unstable angina (UA). CD28^null^ T cells are found in large amounts in the blood of patients who experience these coronary diseases [14]. Flow cytometry was used to detect the landscape of atherosclerotic plaques in patients with UA compared to those with stable angina. The data identified higher median frequencies of CD28^null^ T cells in patients with UA than in patients with stable angina, implying that unstable atherosclerotic plaques are more likely to become invaded by clonally expanded CD28^null^ T cells. Patients with ESRD were also studied, as the leading cause of morbidity in these patients includes cardiovascular disease [66]. Flow cytometry in heparinized venous blood specimens demonstrated that the frequency of CD28^null^ T cells was significantly higher in patients with ESRD than in healthy subjects, indicating that these T cells possess pro-atherogenic properties.

### 3.6. Treg

Treg cells typically secrete atheroprotective cytokines, such as IL-10, which aid in the regulation of T cell-mediated immune responses [67]. Additionally, Tregs may express co-inhibitory molecules such as cytotoxic T-lymphocyte-associated protein 4 (CTLA-4), which downregulates the expression of co-stimulatory molecules, such as CD80/CD86, on APCs and ultimately suppresses conventional T cell activation [68]. The depletion of Tregs, through combined *Cd80/Cd86* genetic deficiencies in mice, aggravates atherosclerosis [32]. The adoptive transfer of Tregs into *ApoE*^−/−^ mice, on the other hand, reduced plaque burden confirming their atheroprotective nature [48].

Treg cells’ atheroprotective functions are fundamentally dependent on the stable expression of the transcription factor Foxp3. The role of Treg cells in atherosclerosis is believed to be atheroprotective but can become pro-atherogenic in the advanced stages of the disease upon the conversion to ex-Treg cells [12]. Researchers observed instability and a decrease in Foxp3 activity upon activation and inflammation in the central nervous system (CNS) in Treg cells that had previously expressed high levels of Foxp3 [69]. Using a cell lineage-tracker mouse line, transcriptomic differences between Tregs and ex-Tregs were analyzed following cell sorting and bulk RNA-seq. Genes that identified ex-Tregs included inflammatory markers such as *Tbx21*, *Ifng*, *Ccl4*, and *Nkg7* compared to *Foxp3* and *Il2ra* for the Treg subset. These results were projected on human scRNA-seq datasets, which allowed for the investigation and validation of surface markers for human exTregs that included CD16 and CD56 [70].

### 3.7. iNKT

Additionally, invariant natural killer T cells (iNKT) are a subset of T cells which may be CD4^−/+^, and they are believed to influence the initial phase and the advanced stages of atherosclerosis by exacerbating and destabilizing the plaque and the necrotic core [71]. iNKT cells can be activated through antigen presentation using the MHC-I-like protein, CD1d, upon lipid fragment presentation. To address their role in atherosclerosis, studies have backcrossed *ApoE*^−/−^ mice with *Cd1d*^−/−^ mice, which led to a reduction in atherosclerosis [72]. Additionally, studies have stimulated iNKTs using a cognate lipid antigen, α-galactosylceramide (α-GalCer), which induces inflammation and increases plaque burden in hyperlipidemic mice [72,73]. Taken together, the current research suggests iNKTs are proatherogenic. However, one study used 1,2-dipalmitoyl-*sn*-glycero-3-phosphoethanolamine-*N*[methoxy(polyethyleneglycol)-350] (DPPE-PEG_350_) as an iNKT lipid antigen, which resulted in reduced novel and established atherosclerosis progression [74].

### 3.8. CD8^+^

Outside of CD4^+^ cells, CD8^+^ T cells mature into cytotoxic T cells and are known for their cytotoxic pathways, typically secreting perforin and granzyme, which help eliminate virally-infected cells as well as cancer cells [12]. In the context of atherosclerosis, CD8^+^ T cells are abundantly found in lesions and have been shown to exhibit pro- and anti-atherosclerotic properties; however, evidence on their role is limited. No change in atherosclerosis was observed in *ApoE*^−/−^*CD8*^−/−^ mice [75]. However, depletion of CD8^+^ T cells in *ApoE*^−/−^ mice led to the improvement of early stage atherosclerotic plaques suggesting a pro-atherogenic role [76,77]. A second study focused on late stage atherosclerosis did not find similar results, but instead demonstrated that the depletion of CD8^+^ T cells led to larger, less stable plaques with increased T_H_1 and macrophage content [78]. Additionally, a recent clinical study demonstrated the role of effector CD8^+^ cells in early vascular wall injury in response to percutaneous transluminal angioplasty (PTA) in patients with peripheral artery disease (PAD). Peripheral blood samples were analyzed before and directly after the PTA, and a significant reduction in immunosenescent and activated effector CD8^+^ cells was observed following the procedure. This was likely due to the adhesion and recruitment of T cell subsets to the injured vascular wall [79].

### 3.9. γδ T cells

Unlike CD4^+^ and CD8^+^ T cells which have TCRs composed of α and β chains, γδ T cells have a TCR consisting of γ and δ chains. Furthermore, γδ T cells do not require antigen presentation for activation [80]. While γδ T cells have been identified in atherosclerotic plaques, their role in the disease progression remains unclear. *TCR*γ^−/−^*/ApoE*^−/−^ mice fed a western diet or a normal diet for 10 weeks demonstrated no change in atherosclerotic plaque size compared to control mice indicating that γδ T cells may not be involved in atherosclerosis [81]. However, γδ T cells can secrete IL-17A, which may blur the line between the functions of T_h_17 cells [82].

## 4. Detection Methods for Lesional T cells

Over the past forty years, advances in isolation procedures and technology have helped uncover new discoveries about T cells as well as potential antigens within pre-clinical and clinical atherosclerosis models. In 1985, T cells were identified in human atherosclerotic plaques using fluorescent microscopy [83]. The study showed that T cells made up 11% of all the cells identified, but macrophages were identified as the predominant cell present within lesions. Follow-up studies were performed to identify T cell subsets, which indicated that CD4^+^ T cells were found mainly in the fibrous cap region [84]. While still performed in a variety of atherosclerosis studies today, immunocytochemistry and fluorescent microscopy present challenges in their ability to subclassify immune cells such as T cells due to limited fluorescent channels for analysis. The development of flow cytometry has allowed for expanded fluorescent channels to analyze cellular populations [85]. Early flow cytometry studies, for example, were used to compare T cell populations and their activation status between plaque and blood samples from patients with atherosclerosis [86]. Here, researchers demonstrated that ~76% of lymphocytes detected in plaque samples were T cells, while ~64% of lymphocytes detected in blood samples were T cells. Compared to peripheral blood, CD8^+^ T cells were slightly enriched in plaques while CD4^+^ T cells were more abundant in blood. Likewise in *ApoE*^−/−^ mice, T cells were observed in healthy aortas, but also expanded 5-fold in an advanced atherosclerosis model following a high fat diet [9]. These results represent more traditional methods of analyzing the cellular composition of atherosclerotic plaques and provide evidence that T cells play a role and could be directed toward specific antigens in plaques (see Table 2).

### 4.1. Transcriptomics and Proteomics

Today, flow cytometry is typically used in combination with novel methods such as RNA-sequencing (RNA-seq) or more specialized methods like TCR-sequencing (TCR-seq) to analyze transcriptomic patterns of immune cells within atherosclerotic plaques [87]. A pivotal study analyzed human atherosclerotic plaques, which confirmed T cells to be the predominant lesional cell accounting for 52% of leukocytes found within plaques [8]. An amo7unt of 14 cell populations, including five subsets of CD4^+^ T cells were identified, which speaks to the cellular diversity in lesions. When analyzing T cell subsets, T_H_1 cells are notably present in atherosclerotic plaques, which likely led to the production of inflammatory cytokines driving the inflammatory pathogenesis [88]. T cell subsets are commonly identified by the proteins they express including transcription factors as well as cytokines; however, clustering within scRNA-seq studies appears to divide T cell subsets based on activation status rather than mRNA expression of lineage-specific identifiers [7,8,88]. The development of mass cytometry (CyTOF) allows researchers to gain proteomic information and apply it to transcriptional information from methods such as single cell (sc) RNA-seq [88]. CyTOF works by using earth metals conjugated to antibodies to detect specific protein markers expressed by cells [89]. CyTOF in combination with scRNA-seq has shown a high correlation between protein expression and transcriptional clusters of cellular populations, including T cells, from mouse aortas [7]. Researchers demonstrated that CyTOF was able to detect underrepresented T cell subpopulations, such as γδ T cells, further demonstrating the cellular diversity within plaques.

Similarly, Cellular Indexing of Transcriptional Epitope Sequencing (CITE-SEQ) has been developed which allows researchers to combine proteomic and transcriptional information to a population of cells [87]. T cells were also identified as the most abundant immune cell present within symptomatic human plaques following an endarterectomy [6]. T cells found in plaques displayed a high expression of the programmed cell death protein (PD-1), indicating more activation and exhaustion than T cells from paired blood samples. The activation of T cells paired with chronic inflammation found within plaques could lead to T cell exhaustion [90]; however, additional models will need to confirm this and evaluate potential treatments.

Compared to traditional methods, scRNA-seq has allowed atherosclerotic plaques of both mice and humans to be analyzed in further detail. Studies utilizing scRNA-seq have shown T cells from human plaques to be the predominant cell present, representing ~65% [6] and ~52% [8] of leukocytes. Whereas T cells from murine aortas have been shown to be more equally distributed between healthy and atherosclerotic models, one study showed similar fractions of T cells in the adventitia between wild type and *ApoE*^−/−^ mice [91]. The differences in proportions of T cells and their respective locations will need to be studied in further models. However, direct comparisons cannot be made between humans and mice due to differences in species as well as genetic modifications implemented in experimental mice models [88,92]. While scRNA-seq has aided in identifying and clustering novel immune cells in lesions, there are key limitations to consider with this method. Like other methods, scRNA-seq requires digestion by various enzymes to obtain cells from tissues, which may cause stress and damage to larger or fragile cells, such as macrophages, and could potentially provide inaccurate results from isolation that may skew population statistics toward T cells [88,93]. Additionally, transcripts for some T cells may be weakly expressed, which can make cluster identification difficult when using traditional flow cytometry identifiers such as cluster markers for T cell subsets [88]. Together, scRNA-seq is being developed as a novel method for analyzing the cellular heterogeneity of atherosclerotic plaques while challenging the knowledge previously obtained from more traditional methods.

Spatial transcriptomics, which combines expression data with anatomical location information, is emerging as another novel method to detect immune cells in lesions while also gaining specific location information within plaques [94,95]. Spatial transcriptomics analyzes the organization of cells in regions of tissues, working to further understand biological function [96]. Recently, three regions (proximal (P), most stenotic (M), and distal (D)) of human atherosclerotic plaques were studied using a spatial method [94]. After performing sectioning within these three locations, results showed the rupture site to be located within the proximal plaque, near the M region. Bulk RNA-seq showed a greater proportion of macrophages, NK, and B and T cells present within the M region compared to the D region. Notably, there were also differences in gene expression markers within plaque regions as spatial transcriptomics showed the gene *MMP9* to be highly expressed in locations of rupture (19.7%) compared to stable plaques (2.7%). Altogether, studying specific rupture site locations has given researchers a better understanding of the inflammatory cells and gene expression markers present, while indicating the need for future studies.

### 4.2. Methods to Study Antigen Specificity

Although T cells and their subsets have been identified within plaques, their antigenic specificity in atherosclerosis remains unclear. T cells are defined by their T cell repertoire, which provides diversity through the rearrangement of V, D, and J genes [97,98]. This diversity allows T cells to recognize a wide range of antigens, and alterations to this repertoire have been shown to result in immune-mediated diseases [99,100]. T cell receptor sequencing (TCR-seq) is the most recent method utilized to study the T cell repertoire in the presence of atherosclerosis. This method is performed by deep sequencing of the TCR to analyze diversity, ultimately giving a better understanding of the immune response present [101].

Currently, studies which utilize TCR-seq to analyze T cells in atherosclerosis are limited. In 2017, a study was performed which used immune repertoire sequencing (IR-Seq) based on next-generation DNA sequencing (NGS) to analyze the TCR-β repertoire in both plaques and peripheral blood samples of patients with atherosclerosis [102]. TCR-β repertoire was reduced in plaques, and V and/or J genes were altered compared to samples from peripheral blood and healthy controls. Although the amount of T cell clonotypes was reduced in plaques, the highest frequency clonotypes were elevated in plaques when compared to peripheral blood samples. This study was the first to identify the clonotypes V29-1J2-1, V20-1J1-6, V6-3J2-7, and V11-2J2-2, which were unique to atherosclerotic plaques. The researchers also found the gene *TCR-V6*, which recognized ox-LDL. Ox-LDL has been identified in previous studies as a potential autoantigen targeted by T cells in atherosclerosis [103,104]. A more recent study focusing on patients with acute coronary syndrome (ACS) used TCR-seq to investigate TCR repertoire in the peripheral blood of patients with varying degrees of disease [23]. Patients with ACS displayed a reduced diversity of the TCR-β CDR3 repertoire compared to patients without ACS [23]. There was also a difference in the overlap of TCR-β clonotypes between ACS and non-ACS patients, showing five TCR-β CDR3 clonotypes found only in the presence of ACS. Together, these were two of the first studies which used TCR-seq to show that autoantigens could drive T cells to different clonotypes, ultimately leading to less diversity of TCR in the presence of atherosclerosis [23,102]. 

Additionally, single cell T cell receptor sequencing (scTCR-seq) has emerged as a method to investigate autoimmune features of T cells in the presence of atherosclerosis [24]. The clonal expansion of T cells within plaques appeared due to CD4^+^ T cells engaging with antigen. Tregs also seemed to engage with antigen and expand, but to a lesser extent. These results indicate that T cells present in plaques engage with autoantigens and expand, commonly seen in autoimmune diseases [24,105]. Previous studies have shown that self-antigens such as apoB could drive the autoimmune response of T cells in atherosclerosis [106,107,108]. Protective apoB^+^ T cells, resembling Treg cells, are present in healthy mice models [108]. However, in atherosclerotic mice models and humans with atherosclerosis, apoB^+^ T cells were increased and shifted from an anti-inflammatory Treg cell to a pro-inflammatory T_H_1/T_H_17 phenotype, suggesting that apoB^+^ T cells can expand to become pathogenic and autoimmune in nature. However, further research will need to be performed to evaluate the autoimmune components of atherosclerosis and their mechanisms. The development of TCR-seq has provided a new way to analyze T cells by targeting their TCRs to gain information based on antigen specific immune responses. While TCR-seq has provided insight into the nature of atherosclerosis and identified a potential autoimmune component, there are limitations when using this method, including sensitivity and cost [24,101].

## 5. Conclusions

Accumulating evidence points to a crucial role of T cells in the development and progression of atherosclerosis, their activation and polarization into pro-inflammatory subsets, such as T_h_1 and T_h_17 cells, contribute to the chronic inflammation observed in atherosclerotic lesions. However, the balance between effector and regulatory T cell subsets further influences atherosclerosis progression with increasing plaque sizes typically observed in instances of low Treg numbers. To gain a comprehensive understanding of T cell involvement in atherosclerosis, various methods have been employed to quantify T cell subsets, including flow cytometry, immunohistochemistry, and transcriptomic analyses. These techniques offer valuable insights into the complex interplay between T cells and their potential antigens in atherosclerosis, with evidence pointing towards an autoimmune component. Targeting T cells holds promise for developing novel therapeutic strategies aimed at mitigating inflammation and hindering atherosclerotic plaque formation. Currently, targeting T cells specifically in atherosclerosis remains a challenge. Clinical trials, including the Canakinumab Antiinflammatory Thrombosis Outcome Study (CANTOS) and Cardiovascular Inflammation Reduction Trial (CIRT), have demonstrated differing results for anti-inflammatory therapies for atherosclerosis [109,110]. Vaccination strategies in murine models have shown success in reducing plaque burden when immunizing against LDL and ApoB [107,111]. Expanding atheroprotective Treg numbers through IL-2 may be a viable therapy; however, no data exists if this protects against cardiovascular disease, as expanded Tregs may result in increased exTreg numbers as well [112]. Continued research efforts in understanding the multifaceted roles of T cells in atherosclerosis are essential for improving diagnostic tools and designing effective immunomodulatory therapies in the future.

## Figures and Tables

**Table 1:** Factors affecting T cell activation and polarization. List of several factors including cognate antigen recognition, co-stimulation, and metabolism and their roles in T cell responses in atherosclerosis.

Factor	Description		Contribution
Cognate Antigen Recognition	Genetics	MHCII	Required for activation of CD4^+^ T cells, loss leads to decreased Tregs and increased atherosclerosis
		HLA-B*13:02	Pro-atherogenic allele
		HLA-C*06:02	Pro-atherogenic allele
		HLA-B*38:01	Atheroprotective allele
		HLA-DRB1	Pro-atherogenic allele
	Antigens	Low density lipoprotein (LDL)	Potential (auto) antigens for T cells associated with atherosclerosis
		Oxidized (ox) LDL	
		Apolipoprotein (Apo) B	
		Heat shock proteins (HSP)	
Co-stimulation	CD80/86-CD28		Deficiency demonstrated less burden, decreased APCs, decreased T_H_1 response, and reduced Tregs
	CD40-CD40L		Deficiency suppressed T_H_1 response leading to reduced IFN-γ and lowered plaque burden
	GITR		Constitutive activation promotes Tregs and reduces atherosclerosis
	CD27		Deficiency led to lowered Treg numbers and increased atherosclerosis
	PD-1		Deficiency enhanced T cell reponses leading to increased atherosclerosis
Metabolism	Oxidative phosphorylation		Primarily used by Tregs
	Glycolysis		Primarily used by effector T cells, but Treg migration can depend on glycolysis. Increased Treg glycolysis can be linked to decreased FoxP3 expression
	Diet		Western diet may reduce T cell proliferation. Naïve T cell numbers decreased dependent on severity of cardiovascular disease

**Table 2:** Methods Used to Detect T cells in Pre-Clinical and Clinical Atherosclerotic Models. List of methods to detect, quantify, and classify T cells in atherosclerosis.

Method	Purpose	Challenges/Limitations
Fluorescent Microscopy	Identify T cells in atherosclerotic plaques. Remains used in a variety of atherosclerosis studies today.	Limited number of fluorescent channels.
Flow Cytometry	Used in many studies to sort cells; provides expanded fluorescent channels for analysis.	Relies heavily on expression of CD markers, which may not match gene expression in transcriptomics data.
scRNA-seq	Can assess cellular heterogenueity of tissue like plaque to cluster T cell subsets based on gene expression patterns.	Weakly expressed transcripts such as lineage-specific transcription factors difficult to analyze, enzymatic digestion of tissue may degrade some cell types, T cells may cluster due to activation status.
CYTOF	Detect specific protein markers expressed by cells, commonly used alongside scRNA-seq. Has shown the ability to detect underrepresented T cells subpopulation.	Does not analyze transcriptional information. Usually used in combination with scRNA-seq.
CITE-SEQ	Combines proteomic and transcriptional information.	Additional pre-clinical studies needed to confirm the methods of CITE-SEQ in analyzing T cells within atherosclerotic plaques.
Spatial Transcriptomics	Combines expression data with anatomical location information to gain specific location information. Used to analyze genes in relation to specific location within plaques.	Low RNA expression in lesional cells, especially necrotic core, may produce low quality data. Requires additional studies on both pre-clinical and clinical models.
TCR-seq	Analysis of TCR enhances ability to detect atherosclerosis specific antigens.	Sensitivity and cost.

## Data Availability

Not applicable.
